# Genomic Impacts of Biological Exposures

**DOI:** 10.3390/jdb14010012

**Published:** 2026-03-05

**Authors:** Amalia S. Parra, Christopher A. Johnston

**Affiliations:** 1 Oak Ridge Institute for Science and Education (ORISE), Oak Ridge, TN 37831, USA; sancham9@unm.edu; 2 U.S. Department of Energy (DOE), Washington, DC 20585, USA; 3 Office of the Director of National Intelligence (ODNI), McLean, VA 22102, USA; 4 Department of Biology, University of New Mexico, Albuquerque, NM 87131, USA

**Keywords:** exposome, exposure biology, transcriptomics, systems biology

## Abstract

Development and maintenance of complex tissues depends on a number of coordinated steps from early development through adulthood. These processes are fundamentally controlled by highly regulated gene expression patterns. Although critical contributors during development, intrinsic changes in gene expression alone cannot fully explain the complicated pathways that control tissue homeostasis. Rather, tissues are continuously exposed to extrinsic factors that also influence essential cellular processes. These external environmental factors are collectively known as the exposome. Notably, how different exposures impact gene expression and protein function, as well as how certain exposures lead to disease states, is not well understood. To understand how internal and external factors influence organismal development and homeostasis, it is necessary to consider how genetic and nongenetic components interact to direct critical biochemical pathways. Doing so presents new avenues for precision medicine, understanding disease progression, identifying biological threats, and improving biological security concerns. In this review, we present recent advances in exposure biology, focusing on how these innovations can help identify novel biomarkers to better understand changing exposome components. We also discuss the need to integrate technologies and exposure research to better identify and predict threats.

## 1. Introduction

Biological systems are highly regulated via internal mechanisms, and these processes are tempered by diverse external stimulation. These environmental factors can elicit positive or negative responses, depending on how they interact with internal biological pathways. The interaction between extrinsic environmental factors and intrinsic biological processes, controlled often at the genetic level, has become an important topic for understanding how biological responses are modulated. Moreover, biological systems can be exposed to different environmental components throughout the lifetime. Technological advances have increased the amount of genomic data produced, helping to identify relationships between genes and diseases. Still, these technologies have been unable to predict disease emergence or disease progression. Additionally, the toxicity of certain exposures may depend on external parameters and the nature of the exposure (length of exposure, timing, and route of exposure). Thus, identification and assessment of potential threats is more effective with an in-depth understanding of how the compilation of exposures influences an individual’s biology. This is important because convergence of different environmental exposures and biological mechanisms may pose unexpected threats. Along with this, assessing and detecting threats using biomarkers and sensors will depend heavily on an understanding of the interplay between exposures and biology, beyond the output of high-throughput methods alone. Here we present advances in exposome research and discuss the molecular and computational approaches that can help advance predictive modeling and longitudinal tracing. While the exposome encompasses all exposures, including social and physical encounters, this review will focus specifically on research advances describing how chemical and biological exposures can impact gene expression and function.

## 2. Exposure Biology and Precision Medicine

**Figure 1:**
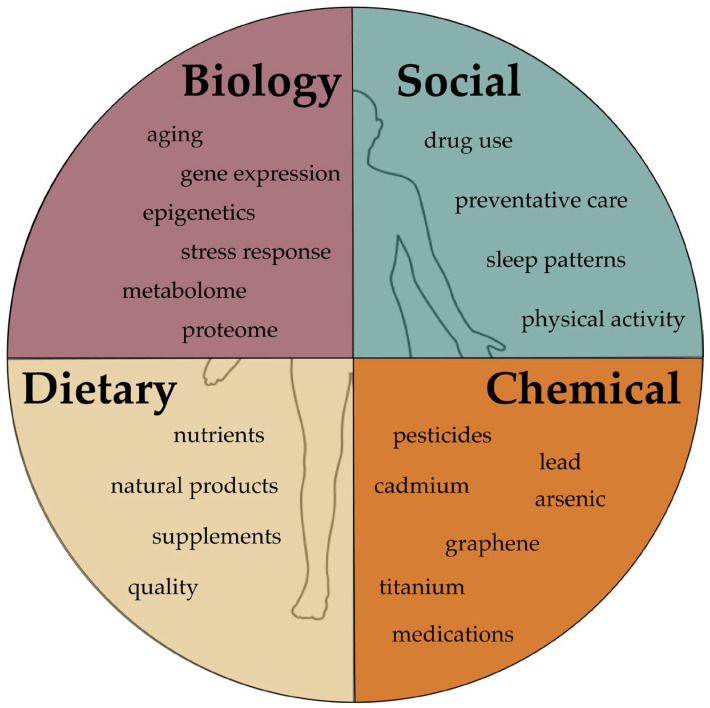
Factors that impact exposure responses and the propensity for disease emergence. Biological, chemical, and dietary variables interact directly with internal biology, while social aspects tend to trigger biological responses indirectly. Biology: Individual variations in gene expression and epigenetic patterns frequently guide the proteome and metabolome. Together, these impact responses to external and internal stimuli, which in turn are influenced by chronological and biological age, and vice versa. These are discussed further in the Section 3. Social: The social environment, relationships, economic status, sleep patterns, and a healthy lifestyle are important modulators of the other aspects of the exposome. Dietary: Food intake (quality, type, etc.) is an important variable that drives individual biological factors including disease risk and aging. Chemical: Exposures may arise from medical interventions, as observed with cadmium, graphene, and titanium, which are components of drug delivery systems, as discussed in the Section 2.2. Pesticides, arsenic, and lead are included in the Section 4.

Individuals are exposed to numerous agents throughout life that cause endogenous changes. The broad exposures an individual experiences throughout a lifetime and the related changes are called the exposome (Figure 1). This concept was first developed to address environmental factors in the study of disease emergence. Studying the exposome, however, has become an important area of research in terms of finding novel ways to better interpret exposome data and understand how the environment interacts with genetics. Innovations in genome sequencing and molecular techniques have led to significant advances in our understanding of genetics, but unfortunately, methods for interrogating the exposome are not as advanced. Devising new and efficient ways to collect and interpret exposome data is critical to understanding how biology responds to different types of exposure. Steps to do so include studying intrachemical interactions, chemicals, genetics, and how a genetic profile influences responses to exposures. These approaches will advance the field of precision medicine, which seeks to incorporate genetics into treatment plans, including drug response analyses.

### 2.1. Pharmacogenomics

Drug treatments, including chemotherapy, are common exposures that elicit biological reactions. Beneficial and toxic responses to therapeutic agents or medications have been widely studied through pharmacogenomics (Table 1). Particularly, differences in drug metabolism and therapeutic effects have been two important areas of interest [1]. This is because polymorphisms, sex, and health history are responsible for much of the variability observed in drug resistance, efficacy, and side effects [2,3]. Enzymes involved in drug metabolism are a source of differential responses to drugs, as are membrane transporters and target modifications. The pharmaceutical industry has relied more on genetics and external exposures to design more effective therapeutics [4]. Advances in sequencing technology have helped identify genomic parameters that influence drug efficacy and side effects. This information has been used to develop novel, improved therapeutics for the treatment of various conditions. For instance, novel agents for the treatment of melanoma have been designed to focus on specific mutations to improve effectiveness and decrease toxicity [5,6].

Beyond drug development, advances in pharmacogenomics and exposure biology have helped elucidate varying responses to widely used drugs, such as warfarin [7,8]. The International Normalized Ratio (INR) is an important parameter used to evaluate bleeding and clotting status for patients on therapies such as warfarin. Maintaining therapeutic levels of warfarin is highly dependent on the INR and is needed to prevent thrombosis or hemorrhage, which can occur if inappropriate levels of the drug are reached. The liver enzyme CYP2C9 is responsible for warfarin metabolism, and its expression levels thus directly impact therapeutic levels. Similarly, drugs that decrease or increase the metabolic function of this enzyme also impact drug levels. Considering that warfarin is a widely used anticoagulant, and that CYP2C9 genetic variations can function as inducers or inhibitors, these parameters may impact treatment outcomes. The evidence on whether genetic considerations will improve warfarin therapy is still unclear. Of the studies performed, each has had opposing conclusions on the value of coupling genetic analysis with current warfarin dosing practices. While one study found that genetically guided dosing decreased the incidence of excessive warfarin doses, another study found no benefit from dosing according to CYP2C9 variations, but noted a significant difference in INR based on race [9,10]. This latter point is important, because a more recent study found that some variations in allele frequencies found in Hispanic/Latino populations impacted warfarin dosing [11]. While these findings are inconclusive regarding the benefit of genetically guided warfarin treatment strategies, they may better inform initial dosing regimens and point to the continued need to explore genomic impacts on drug efficacy.

Treatments for autoimmune conditions such as rheumatoid arthritis have also made use of genomic advances to study polymorphisms that may influence monoclonal antibody efficacy and overall outcomes of available therapeutics [12]. Monoclonal antibodies like adalimumab and infliximab are included in treatment regimens for numerous autoimmune conditions. Treatment outcomes include a mix of initial good responses, with responses waning over time, and good responses followed by emergence of resistance or adverse side effects [13]. Genetic variations at the HLA locus have been highlighted as probable elements that alter treatment outcomes [14,15]. In one cohort, carriers of HLA-*05:05DQA1 developed anti-drug antibodies earlier, following treatment with infliximab or adalimumab [16]. Previous studies have demonstrated that even small amounts of a drug, such as infliximab, are enough to trigger antibody production. Concomitant with these findings, other studies have found similar responses in pediatric patients. Rather than HLA differences being responsible for differential drug effects, neutrophil CD64 (nCD64) seems to be the primary factor responsible for decreased infliximab effects in a pediatric population [17]. Production of drug-clearing antibodies may explain the biphasic response to these biologics and onset of adverse side effects. Methylation patterns are avenues that may have a role in longitudinal changes in response. Fluctuating methylation patterns have been noted following treatment with anti-TNF biologics. To no surprise, these methylation changes involve genes responsible for immune system and immune response regulation, hematopoietic stem cell development, and lymphoid organ development [18]. Unlike previous analyses examining decreased anti-TNF efficacy, these differential methylation patterns correlated with other exposome-relevant areas, including body mass index and smoking history [18,19].

Early drug design and development strategies focused on developing interventions for various conditions. While the goal is still to design effective treatments, the referenced studies emphasize the need to further investigate how the information gathered through research can be harnessed to improve drug responses and design more effective treatment plans. This information has already proven to be valuable for determining dosage for widely used drugs such as beta-blockers (metoprolol) [20]. Continued use of individual exposures and genetics to guide treatments and development of more effective agents is key to advancing personalized medicine.

**Table 1:** Pharmacogenomics and precision medicine.

Compound Name	Uses	Genetic Associations; Gene (SNP)	Clinical Impact	Refs.
Warfarin	Anticoagulation, venous thrombosis, pulmonary embolism	VKORC1 (c.-1639: G > A) CYP2C9 (alele: 2, 3, 5, 6, 8, 11, 12, 13, 15) CYP4F2 (rs12777823)	Increased sensitivity Reduced clearance Increased sensitivity	[21,22,23,24,25]
Adalimumab	Rheumatoid arthritis, ankylosing spondylitis, Crohn’s disease, ulcerative colitis, hidradenitis suppurativa, psoriatic arthritis, plaque psoriasis	Tumor necrosis factor (rs1800629) FCGR2A (rs1801274-GG) ATG16L1 (rs2241880) IL-6 (rs1800795: GG > CC) MAP2K6	Increased drug response Decreased drug response Decreased drug response Increased drug response Increased drug response	[12,26,27,28,29]
Infliximab	Crohn’s disease, ulcerative colitis, rheumatoid arthritis, ankylosing spondylitis, psoriatic arthritis, plaque psoriasis	HLA-B*39:01, B*08:01 HLA-C*12:03 HLA-DPB1*10:01 HLA-DQB1*02:01 HLA-DRB1*04:04	Drug-induced liver injury Drug-induced liver injury Drug-induced liver injury Drug-induced liver injury Anti-drug antibody formation	[30,31,32,33,34]
Etanercept	Rheumatoid arthritis, psoriasis, psoriatic arthritis, ankylosing spondylitis	HLA-DRB1 *04:01 TNF-⍺ (rs1800629: AA > GG)	Increased drug response Decreased drug response	[35,36,37,38]
Temozolomide	Squamous cell carcinomas of the head and neck, glioblastoma	RYK (rs4470517: AA > GG) O-6-methylguanine-DNA methyltransferase (MGMT) (rs2308321-G) Isocitrate dehydrogenase (IDH) Peptidylarginine deiminase 4 (PADI4) Spliceosome-Associated Protein 4 (SDF4) Tumor Protein p53 Inducible Transcript 1	Higher drug sensitivity Decreased drug response Increased drug resistance Increased drug resistance Increased drug resistance Increased drug resistance	[39,40,41,42]

### 2.2. Nanomedicine and Drug Delivery Systems

Nanomaterials have become an important part of advanced manufacturing techniques, diagnostics, and novel drug delivery systems. Efficient drug delivery and tissue targeting are two benefits of nanomedicine; however, they also present additional areas for consideration. Increased use of these is beneficial but also presents a new source of potential health threats. The fate of nanomaterials after they have performed their therapeutic functions is not well understood, beyond knowledge that they accumulate in tissues for unknown periods of time. Epigenetic and transcriptomic changes can occur anytime following exposure and often include complex changes in gene expression [43]. Apart from exerting therapeutic effects on target tissues, nanomaterials can induce molecular changes in other tissues they encounter. As the central detoxification and waste excretion unit in the body, the renal system is an essential tissue for studying nanomaterial–tissue interactions. Nanomaterials can interact with glomeruli or proximal tubules of the renal system during excretion [44]. Elimination of nanomaterials of different sizes may injure the glomerular podocytes, lead to loss of brush borders on tubular cells, and cause necrosis [45]. Apart from structural damage to the overall architecture, molecular changes in response to nanoparticles—namely, increased expression of cell cycle genes (*CCND1*) and genes involved in immune responses (*TNF*, *CSF2*, and *GDNF*), predominantly inflammation (*Il-18*)—also occur [46]. These changes in gene expression are mediated by accompanying alterations to epigenetic patterns, with a large number of cancer-associated genes (VEGF and PI3K-Akt signaling) exhibiting hypomethylation [46]. Aberrant signaling of lysine demethylase 6A (*KDM6A*) and 6B (*KDM6B*), two H3K27 demethylase enzymes involved in cell differentiation, organ development, and tumor growth, was also observed in these tissues [46], suggesting an inflammatory response to exposure [47]. In the long term, these inflammatory and growth dysregulation responses may lead to pathologies, including metabolic dysfunctions preventing energy production and increased accumulation in already diseased tissues [48,49].

Murine studies analyzing the response to gold nanoparticle (NP) exposure showed increased methylation in promoter regions in genes responsible for cell cycle regulation (cyclin dependent kinase and *trp53*) and protection from oxidative stress (glutathione reductase) in lung tissue [50]. Further changes are seen at the RNA level: recent studies have identified differentially regulated RNAs that are associated with double-stranded break repair via non-homologous end joining, transcriptional activity in cancer, and stem cell pluripotency [51]. Interestingly, methylation patterns differ across nanomaterial composition, size, and concentration—pointing to the widespread interaction of these particles within biological systems [48]. These findings raise questions about the clinical implications of nanomaterials and nanomedicine. First, persistence of reactive oxygen species can render systems more susceptible to pathologies and decreased efficiency, particularly in situations in which ROS homeostasis is disturbed. Second, nanomaterials can accumulate and cause chronic disease. Third, some nanomaterials have the potential to reach the brain, where they can contribute to neurodegeneration and immune activation [48]. Apart from causing changes to exposed tissue, nanomaterial exposure can induce heritable epigenetic changes consistent with cardiac dysfunction [52]. Mechanisms supporting pathologic genetic changes comprise alterations in histone dynamics, specifically H3K27ac and H3K4me1, depending on the exposure [53]. One important parameter to consider is the dose and timing of exposure required to elicit epigenetic changes. Studies using sublethal concentrations of titanium dioxide (TiO_2_) or Zinc oxide (ZnO) in lung fibroblasts demonstrated that treatment with these nanomaterials caused widespread decreases in DNA methylation and methyltransferase activity [54]. Further, studies examining multiple human cell lines, including human colorectal (Caco-2) cells, found similar epigenetic responses [55]. Heavy metal toxicity has been widely studied; conversely, the potential toxic outcomes of nanomaterial exposure are not as well understood.

Considering the ability to detect epigenetic changes using existing technologies and the pace at which novel technologies are introduced, the future of new biomarker identification and methods to predict responses is promising. Despite accumulating evidence signifying nanomaterial toxicity, nanoparticles have been used to address many existing conditions, including glomerulonephritis seen in diabetes and fibrosis in chronic kidney disease, and prevent aberrant DNA methylation patterns [56,57,58]. Overall, the field of nanomedicine has advanced enough to be able to use its technology to advance medical sciences. In doing so, new challenges in the form of adverse side effects have also emerged and are significant barriers to using the technology more widely. As briefly discussed here, the benefits of addressing debilitating conditions like diabetic nephropathy are tremendous; however, doing so risks interfering with other biological processes. Nonetheless, the benefits of this technology are clear. Further work elucidating how composition, size, and concentration of nanomaterials impart adverse or beneficial outcomes, and how pre-existing conditions impact responses to treatment, will be fundamental to realizing nanomedicine’s great potential.

## 3. External Exposures and Development

Early life exposures can be more deleterious than exposures later in development and adulthood due to the highly complex mechanisms that occur in utero and during early stages after birth [59]. These critical periods establish the foundational cell populations that are responsible for creating specialized tissues in the developed organism. This is true across different organisms, including humans, flies, and worms [60]. These conserved mechanisms involve close coordination of cell growth and proliferation with cell specialization. While internal errors can cause developmental defects, existing research suggests that external factors may influence the likelihood that these errors result in disease states. For instance, structural defects of the spine, such as scoliosis, have been attributed to errors in Notch signaling. Exposure to hypoxic conditions, however, exacerbates this spinal defect and causes errors in other signaling pathways that regulate development, such as FGF and Wnt [61]. Synergy of genetic risk factors and environmental toxicants also explains the widespread impact of Bisphenol A. Aftereffects of BPA include extensive adverse changes, including altered vasculature function in mice during pregnancy due to BPA’s prevention of the relaxation of human umbilical arteries (HUA) [62]. Further, expression of genes involved in trophoblast invasion (*TIMP-1*, *TIMP-2*, *MMP-2*, *MMP-9*, *WNT-2*, and *β-catenin*) are misregulated following BPA exposure [63]. In utero exposure to BPA is also linked to birth complications, miscarriage, and low birth weight [64]. In particular, BPA exposure decreases expression of transcripts involved in cell cycle regulation, including CDK1, CDK2, Cyclin B, and PCNA, ultimately resulting in cell cycle arrest [65]. This is problematic because highly mitotic tissues, like nervous tissue, are at risk for BPA-induced defects. Embryonic tissues are particularly susceptible because differentiation is a highly coordinated process that relies on multiple inter-connected steps. Moreover, the rapid rates of growth and development leave little opportunity to repair errors. Additionally, the high energy expenditure during early development also limits repair capabilities. Recent studies in mice reveal specific changes in the expression of genes involved in neurological disease and injury in response to common environmental doses of BPA [66]. More precisely, BPA metabolites alter DNA methylation patterns, changes that later may present as abnormal birth weight and defects in cognitive development [67,68]. Health impacts of BPA have been well studied and were briefly discussed here. Additional exposures of concern, which require more studying, can be found in Table 2.

Interruption of normal cell growth functions also occurs with exposure to other toxicants, like mycotoxins. Found in contaminated crops, mycotoxins are fungal products that can cause acute and chronic toxicity affecting various tissues [69]. The most well-known mycotoxins are Aflatoxins, patulin, ochratoxins, Alternaria toxins, trichothecenes, and deoxynivalenol. Both Aflatoxins and ochratoxins can be found in breast milk, and ochratoxins have also been detected in urine samples [70,71]. Aflatoxins are well-known carcinogens metabolized by cytochrome P450 enzymes to produce toxic metabolites. Toxicity includes production of DNA adducts that interfere with replication and transcription machinery [72]. This leads to errors in DNA damage repair and mutagenesis. Specific outcomes of Aflatoxin exposure during critical developmental stages have recently been demonstrated in *C. elegans.* Highly proliferative germline cells (GCs) in *C. elegans* are responsible for growth and development and are prominent targets of this toxin. Aflatoxin treatment induces increased apoptosis in GCs via elevated expression of genes involved in cell cycle arrest, including *egl-1* and *hus-1* [73]. In humans, Aflatoxins passed from mother to fetus cause dysfunction in insulin and growth hormone signaling and errors in developmental processes, including low birth weight [74].

The mycotoxin ochratoxin A (OTA) interferes with human embryonic cell survival and proliferation by activating apoptosis and promoting oxygen stress [75]. Its presence in amniotic fluid is correlated with a large proportion of fetuses with genetic defects [76]. After birth, infants exposed to Aflatoxin exhibit low birth weight and diminished head size [77]. Children are highly vulnerable to biotoxins due to underdeveloped detoxification mechanisms. This is partly due to the inexperienced immune system in young children. In some cases, exposure to Aflatoxin in utero results in differential DNA methylation patterns [78]. Specific effects of OTA exposure also depend on health history, age, gender, class of toxin, and pharmacogenomics (Table 2) [79]. Studies in *Danio rerio* have demonstrated that exposure to OTA promotes mitochondrial malfunction due to errors in detoxification and antioxidant mechanisms [80]. Specifically, OTA interferes with the cellular response to oxidative stress by decreasing expression of transcription factors involved in activation of these pathways. Further, detoxification is also hindered via reduced expression of enzymes responsible for clearing toxic metabolites, with kidneys being intensely susceptible [81,82]. Previous studies have highlighted the epigenetic response to various exposures and pointed to embryonic genome modulation and heritable DNA modifications that impact development [83]. In murine studies, OTA impairs hippocampal cell survival and induces toxicity in neuronal cells [84]. More details regarding mycotoxin toxicity remain to be elucidated. Notably, the detoxification mechanisms of many mycotoxins have not been identified. The impacts of length and route of exposure, resulting genetic changes, and the ability to maintain homeostasis following exposure are also poorly understood.

As a normal part of development, biological systems lose some ability to maintain efficiency and homeostasis. Biological systems age, and this likely influences internal biology–environment interactions. Encounters with chemicals result in cellular responses that vary with biological and chronological aging. Alternatively, various encounters can induce mechanisms consistent with aging or premature aging [85]. Common aging principles conserved across different organisms include decreased signaling mechanisms and disturbance of homeostasis [86]. Early life exposures are principally responsible for biological aging. This presents as epigenetic aging, a series of changes associated with the normal progression of cell senescence, death, self-renewal, and regulation of genetic material [87,88]. Some exposures have been shown to accelerate biological aging and increase disease susceptibility and onset of chronic diseases [85,89]. Building evidence suggests that a summation of exposures determines the degree and rate of decline. For instance, the hallmark endothelial dysfunction observed in cardiovascular disease is attributed to exposure to byproducts from burning plastics and fuels, as well as contact with particulate matter or metals (Table 2) [90,91,92]. These compounds are linked to premature aging that contributes to development of chronic diseases (Table 2) [93]. While aging is an inevitable process, understanding the interplay between genetics and exposures can aid in promoting healthy aging and mitigating the impacts of chronic diseases.

## 4. Genetics and Chemical Exposures

Chemical toxicants are another source of stimulation leading to adverse biological responses. Innovations in genetics and sequencing technologies have identified various genetic profiles associated with disease and treatment outcomes (Table 1). Nonetheless, some individuals exhibit incomplete penetrance for long periods of time, demonstrate variable expression or never present with associated clinical symptoms. These phenotypic variations have been attributed to synergy of multiple genes and interactions with nongenetic factors such as external exposures (Table 2). Individuals can be carriers of disease-associated genes or mutations, but this does not always result in disease symptoms. For example, although numerous polymorphisms and alleles have been linked to multiple sclerosis (MS), the specific trigger for the disease is not known [94]. Potential triggers for MS are believed to arise from imbalances in gut bacteria due to certain exposures and genetic susceptibility [95]. Recent studies have presented associations between chemical exposures and genetic associations that are linked to the onset of MS [96,97]. The molecular basis of these interactions suggests that exposure to certain chemicals causes upregulation of genes associated with MS, such as IL-1β, IL-6, and NFKβ, contributing to immune system dysregulation and thus the hallmark immune-mediated destruction of myelin [98]. Upregulation of these genes often mirrors the pathological state observed in MS. For example, nervous tissue samples collected post-mortem were shown to contain a combination of heavy metals that initiate demyelination. However, not all tissues with toxic metal buildup exhibited MS [99]. Other nervous system disorders such as amyotrophic lateral sclerosis (ALS) and Parkinson’s are attributed to gene–environment interactions [100]. Formerly believed to have solely a genetic basis, Parkinson’s and ALS are now linked with environmental exposures. Emerging evidence indicates that early gastrointestinal dysfunction occurs long before motor and cognitive symptoms of Parkinson’s [101]. More specifically, stress signaling pathways and homeostatic mechanisms in the gut are targets of toxicants such as lead and organophosphates [102,103]. Prolonged lead exposure in rats has been shown to have detrimental effects on antioxidant function, causing motor neuron degradation as a result of increased reactive oxygen species (ROS) production that causes DNA damage, and aberrant protein architecture [104]. These toxic exposures interfere with the intact GI barriers that prevent leakage of contents and death of beneficial bacteria. Together, these cause systemic inflammation, importantly, in immune-privileged areas such as the central nervous system. One important question is whether these events stem from one initiating factor such as gut dysbiosis, or if protein aggregation, sleep disturbances, or other inciting events are required. Individually, gut dysbiosis, protein aggregation, and exposure to toxicants have all been linked to neurodegenerative conditions. Cases of cognitive dysfunction preceding GI disturbances or completely lacking GI symptoms have occurred and raise further questions about the true origins of these diseases [105].

Organophosphates are common insecticides that target acetylcholinesterase in humans and insects. Even at acute levels, organophosphate exposure has been shown to have adverse effects on the nervous system. These effects are often observed in a subset of events involving Paraoxonase (PON1), an enzyme involved in metabolism of organophosphates [106]. Polymorphisms of PON1 direct expression levels and influence the rate of metabolism, ultimately impelling detoxification efficiency. In mice, low activity of PON1 rendered them susceptible to organophosphates while knockout of PON1 resulted in considerably elevated toxicity [107]. The mechanism of toxicity is thought to include organophosphate’s modulation of nuclear receptors and causes oxidative stress, increased inflammation, and decreased levels of PON1 mRNA [108].

These studies point to the importance of gene–environment interactions and how they influence pathology, suggesting that while disease profiles may exist, they might not manifest unless triggered. The time between exposure and manifestation of environmental exposures leading up to clinical presentation could depend on the offending agent. Table 2 provides more information about agents and toxicants that have the potential to cause significant harm but require more research. Future studies will help clarify these and other important unresolved questions about the nature of gene–environment relationships.

## 5. Synthetic Biology and the Exposome

Advances in synthetic biology have furthered understanding of life at the molecular level and have transformed perspectives on biosecurity. The notion of the exposome is even more important in synthetic biology because of the degree of uncertainty that accompanies new technologies. The ability to modify molecular properties of genes and gene products has advanced synthetic biology. This has contributed to a sophisticated understanding of life at the molecular level. Nonetheless, this has also expanded the adversary applications of synthetic biology. Development of synthetic antibiotics to counteract emerging resistance, gene therapies to replace or repair defective genes, and artificial enzymes for improved performance are examples of advantageous applications of synthetic biology [109,110,111,112]. In its infancy, synthetic biology was not widely used due to the limited availability of tools, their complexity, and the associated costs. More recently, implementing molecular engineering techniques and devising new approaches to accelerate development of novel biological materials have become more affordable and accessible [113]. These advances have been impactful but also pose potential threats due to security and health concerns associated with engineered materials [114,115]. Here we discuss the need for continued research to understand how new or modified materials interact with the exposome.

Most of the diseases presented here have a genetic basis, and recent innovative genetic advances have been used to address genetic defects in research settings. These approaches have been successful in congenital heart disease modeling in *Drosophila*, lung adenocarcinoma, and elsewhere [116,117]. Some success has also been observed with attempts to treat rare genetic conditions, as was seen in the treatment of carbamoyl-phosphate synthetase 1 (CPS1) deficiency and approval of agents to treat some blood disorders [118]. Both are examples of the benefits of advancing synthetic biology; however, the technology is not unproblematic. Targeting and delivering the compounds that make these outcomes possible triggers immunogenic or adverse side effects in recipients. For example, commonly used delivery vessels are adeno-associated viral vectors, and the nucleases are from *Staphylococcus aureus* and *Acidaminococcus*. The viral vectors and nucleases are known to trigger immune cell activation, especially in sensitized recipients [118]. Apart from failed therapy, these responses contribute to additional immune activation that may exacerbate disease states. Beyond developing targeted genome editing treatments, future work should also consider the biological impacts of delivering these materials to tissue, namely, the path that these delivery receptacles take to reach tissues, potential off-targets, and the ability to cause more harm through unnecessary activation of immune responses. It is important to remain cognizant of these applications, which have the potential to advance science but also cause substantial harm in the process.

**Table 2:** Select biotoxins of concern. Agents were elected based on their potential to cause harm, severity of health impacts, and ease of access or exposure.

Chemical/Agent Name	Biological Significance	Sources of Exposure	Refs.
Amanitin	hepatic cell apoptosis, oxidative stress	death cap mushroom	[119,120]
Ammonium nitrate	nephritis, methemoglobinemia	fertilizer, explosives, household cleaning products	[121,122]
Benzene	myelotoxicity, hepatotoxicity, inhibition of mDNA synthesis, endocrine disruption, skin cancers	petroleum refining, coal burning, detergents, paint, solvents	[123,124]
Bisphenols	endocrine disruption, oxidative stress, neurotoxicity, apoptosis, hepatoxicity	packaging, production of polycarbonate plastics, synthesis of epoxy resins, diet, dental sealants	[125,126]
Carbon nanotubes	formation of superoxides and reactive oxygen species, mutagenicity, tissue engineering	drug delivery, biosensors, cancer therapy	[127,128]
Carbon tetrachloride	nephrosis, pulmonary edema, dyspepsia, anemia, hepatotoxicity, nephritis, CNS depression, death	refrigerants, cleaning materials, construction materials, fire extinguishers, dry cleaning materials	[129,130]
Crimean–Congo hemorrhagic fever virus	myalgia, neck stiffness, photophobia, petechial rashes, hepatomegaly, splenomegaly, prolonged prothrombin and activated partial prothrombin time, disseminated intravascular coagulation	ticks (*Hyalomma*), infected animals (cattle, sheep, ostriches), contaminated body fluids	[131,132,133]
Hexabromocyclododecanes	highly lipophilic, neurotoxicity, thyroid and breast cancers, thyroid toxicity, cardiovascular toxicity, decreased cell viability, inflammation, transplacental	textiles, building insulation, flame retardants, contaminated water, textile products, dust, contaminated food	[134,135,136]
Hydrogen cyanide	inhibition of cellular respiration, hypoxia, rapid absorption via skin, GI, and respiratory tracts, hemodynamic instability, cardiovascular system toxicity, seizures, nausea, vomiting, coma, confusion, neuropathy, death	combustion of wools, silk, and nitrogen polymers; industrial fires; pesticides; photography; metal processing; electroplating	[137,138]
Lewisite (dichloro(2-chlorovinyl) arsine)	vesicant, multiorgan damage, mucous membrane toxicity, Lewisite shock, erythema, blindness, respiratory failure, enzyme toxicity: pyruvate dehydrogenase, glutathione, acute kidney injury	chemical warfare	[139,140,141]
Mustard agents	vesicant, carcinogen, DNA damage, free radical generation, alkylating agent, inhibition of cellular respiration, cell death	occupational exposures on military sites, previous warfare sites	[142,143,144,145]
Ochratoxin A	nephrotoxicity, hepatotoxicity, teratogenic, formation of DNA adducts	contaminated beverages, wheat, corn, meat products, or fruit	[146,147,148,149]
Organophosphate agents	neurotoxins, cholinergic toxicity, respiratory failure	flame retardants, plasticizers, pesticides, herbicides, warfare	[150,151]
Phosgene	damage to the respiratory tract, pulmonary edema	polycarbonate and polyurethane manufacturing, pesticides	[152]
Polybrominated diphenyl ethers	endocrine disruption, carcinomas	flame retardants, construction materials, household products	[153]
Ricin	ribosome inactivation, cell death, organ necrosis	castor beans, *Ricinus communis* (castor oil plant)	[154,155,156]
Saxitoxin	neurotoxin, ion channel dysfunction	marine/freshwater algae and cyanobacteria	[157,158]

## 6. Conclusions

The ability to predict disease development and the impacts of environmental exposures are important factors in predicting biological catastrophe and advancing human health. While these are certainly beneficial to human health, some have questioned the ethics behind exposome research. Much of the controversy arises from the data collection necessary to establish databanks. Biobanks store data that inform professionals about genes, health, and the environment, and, in turn, help them predict disease risk. Individuals agree to share sensitive information, like DNA and electronic health records, that is used to identify novel genetic variants associated with disease or disease risk [159]. Formation of biobanks with large amounts of data from many individuals certainly provides a benefit for disease assessment and threat analyses. However, more research on ethical considerations is needed. Further discussion of the ethics of exposome research and the establishment of biobanks can be found in recent academic publications [160,161,162]. Identifying additional details about the impacts of exposome exposures, such as those listed in Table 2, on gene expression and the consequences to cell function, along with leveraging this knowledge to devise novel detection and treatment strategies, will be important future research goals.

## Data Availability

No new data were created or analyzed in this study.

## References

[1] Pristner M., Warth B. (2020). Drug-Exposome Interactions: The Next Frontier in Precision Medicine. Trends Pharmacol. Sci..

[2] Waxman D.J., Holloway M.G. (2009). Sex differences in the expression of hepatic drug metabolizing enzymes. Mol. Pharmacol..

[3] Powell N.R., Liang T., Ipe J., Cao S., Skaar T.C., Desta Z., Qian H.R., Ebert P.J., Chen Y., Thomas M.K. (2023). Clinically important alterations in pharmacogene expression in histologically severe nonalcoholic fatty liver disease. Nat. Commun..

[4] Rushing B.R., Thessen A.E., Soliman G.A., Ramesh A., Sumner S.C. (2023). The Exposome and Nutritional Pharmacology and Toxicology: A New Application for Metabolomics. Exposome.

[5] Goey A.K., With M., Agema B.C., Hoop E.O., Singh R.K., van der Veldt A.A., Mathijssen R.H., van Schaik R.H., Bins S. (2019). Effects of pharmacogenetic variants on vemurafenib-related toxicities in patients with melanoma. Pharmacogenomics.

[6] Ibrahim M., Illa-Bochaca I., Jour G., Vega-Saenz de Miera E., Fracasso J., Ruggles K., Osman I., Schober M. (2025). NF1 Loss Promotes EGFR Activation and Confers Sensitivity to EGFR Inhibition in NF1 Mutant Melanoma. Cancer Res..

[7] Agrawal S., Heiss M.S., Fenter R.B., Abramova T.V., Perera M.A., Pacheco J.A., Smith M.E., Rasmussen-Torvik L.J., George A.L. (2020). Impact of CYP2C9-Interacting Drugs on Warfarin Pharmacogenomics. Clin. Transl. Sci..

[8] Qahwaji R., Ashankyty I., Sannan N.S., Hazzazi M.S., Basabrain A.A., Mobashir M. (2024). Pharmacogenomics: A Genetic Approach to Drug Development and Therapy. Pharmaceuticals.

[9] Pirmohamed M., Burnside G., Eriksson N., Jorgensen A.L., Toh C.H., Nicholson T., Kesteven P., Christersson C., Wahlstrom B., Stafberg C. (2013). A randomized trial of genotype-guided dosing of warfarin. N. Engl. J. Med..

[10] Kimmel S.E., French B., Kasner S.E., Johnson J.A., Anderson J.L., Gage B.F., Rosenberg Y.D., Eby C.S., Madigan R.A., McBane R.B. (2013). A pharmacogenetic versus a clinical algorithm for warfarin dosing. N. Engl. J. Med..

[11] Steiner H.E., Giles J.B., Patterson H.K., Feng J., El Rouby N., Claudio K., Marcatto L.R., Tavares L.C., Galvez J.M., Calderon-Ospina C.A. (2021). Machine Learning for Prediction of Stable Warfarin Dose in US Latinos and Latin Americans. Front. Pharmacol..

[12] Lim S.H., Kim K., Choi C.I. (2022). Pharmacogenomics of Monoclonal Antibodies for the Treatment of Rheumatoid Arthritis. J. Pers. Med..

[13] Gagnon A.L., Beauchesne W., Tessier L., David C., Berbiche D., Lavoie A., Michaud-Herbst A., Tremblay K. (2021). Adalimumab, Infliximab, and Vedolizumab in Treatment of Ulcerative Colitis: A Long-Term Retrospective Study in a Tertiary Referral Center. Crohn’s Colitis 360.

[14] Culmsee-Holm F.B., Buhl E., Kraaer M.T., Steenholdt C., Ainsworth M.A. (2025). Identifying Genetic Factors Influencing the Development of Anti-Drug Antibodies in Inflammatory Bowel Disease: A Scoping Review. J. Inflamm. Res..

[15] Liao H., Al-Hillawi L., Neary E., Camesella Perez B., Bedke N., Brain O., Satsangi J., Ternette N., OASIS (2025). OP06 HLA-DQA1*05:01 is associated with loss of response to infliximab, and HLA DQA1*05:05 with loss of response to adalimumab in the Oxford OASIS study. J. Crohn’s Colitis.

[16] Hodges P., Roberts C., Parkes M., Kennedy N.A., Goodhand J., Ahmad T., Consortium P. (2025). Clinical utility of pre-treatment four-digit-resolution HLA genotyping to guide anti-tumor necrosis factor choice and concomitant immunomodulator use. J. Crohn’s Colitis.

[17] Colman R.J., Xiong Y., Mizuno T., Hyams J.S., Noe J.D., Boyle B., D’Haens G.R., van Limbergen J., Chun K., Yang J. (2022). Antibodies-to-infliximab accelerate clearance while dose intensification reverses immunogenicity and recaptures clinical response in paediatric Crohn’s disease. Aliment. Pharmacol. Ther..

[18] Lin S., Hannon E., Reppell M., Waring J.F., Smaoui N., Pivorunas V., Guay H., Chanchlani N., Bewshea C., Bai B.Y.H. (2023). Whole Blood DNA Methylation Changes Are Associated with Anti-TNF Drug Concentration in Patients with Crohn’s Disease. J. Crohn’s Colitis.

[19] Zhang H., Kalla R., Chen J., Zhao J., Zhou X., Adams A., Noble A., Ventham N.T., Wellens J., Ho G.T. (2024). Altered DNA methylation within DNMT3A, AHRR, LTA/TNF loci mediates the effect of smoking on inflammatory bowel disease. Nat. Commun..

[20] Dean L., Kane M., Pratt V.M., Scott S.A., Pirmohamed M., Esquivel B., Kattman B.L., Malheiro A.J. (2012). Metoprolol Therapy and CYP2D6 Genotype. Medical Genetics Summaries.

[21] Kaminsky L.S., Zhang Z.Y. (1997). Human P450 metabolism of warfarin. Pharmacol. Ther..

[22] Dean L., Pratt V.M., Scott S.A., Pirmohamed M., Esquivel B., Kattman B.L., Malheiro A.J. (2012). Warfarin Therapy and VKORC1 and CYP Genotype. Medical Genetics Summaries.

[23] Pratt V.M., Cavallari L.H., Del Tredici A.L., Hachad H., Ji Y., Kalman L.V., Ly R.C., Moyer A.M., Scott S.A., Whirl-Carrillo M. (2020). Recommendations for Clinical Warfarin Genotyping Allele Selection: A Report of the Association for Molecular Pathology and the College of American Pathologists. J. Mol. Diagn..

[24] Patel S., Singh R., Preuss C.V., Patel N. (2025). Warfarin.

[25] Hirsh J., Fuster V., Ansell J., Halperin J.L., American Heart Association/American College of Cardiology Foundation (2003). American Heart Association/American College of Cardiology Foundation guide to warfarin therapy. J. Am. Coll. Cardiol..

[26] Ellis C.R., Azmat C.E. (2025). Adalimumab.

[27] Marquez Pete N., Maldonado Montoro M.D.M., Perez Ramirez C., Martinez Martinez F., Martinez de la Plata J.E., Daddaoua A., Jimenez Morales A. (2021). Influence of the FCGR2A rs1801274 and FCGR3A rs396991 Polymorphisms on Response to Abatacept in Patients with Rheumatoid Arthritis. J. Pers. Med..

[28] Zapata-Cobo P., Salvador-Martin S., Velasco M., Palomino L.M., Clemente S., Segarra O., Moreno-Alvarez A., Fernandez-Lorenzo A., Perez-Moneo B., Montraveta M. (2023). Polymorphisms indicating risk of inflammatory bowel disease or antigenicity to anti-TNF drugs as biomarkers of response in children. Pharmacol. Res..

[29] Dávila-Fajardo C.L., Márquez A., Pascual-Salcedo D., Moreno Ramos M.J., García-Portales R., Magro C., Alegre-Sancho J.J., Balsa A., Cabeza-Barrera J., Raya E. (2014). Confirmation of -174G/C interleukin-6 gene promoter polymorphism as a genetic marker predicting antitumor necrosis factor treatment outcome. Pharmacogenet. Genom..

[30] Jan Z., El Assadi F., Velayutham D., Mifsud B., Jithesh P.V. (2025). Pharmacogenomics of TNF inhibitors. Front. Immunol..

[31] Bruno C.D., Fremd B., Church R.J., Daly A.K., Aithal G.P., Bjornsson E.S., Larrey D., Watkins P.B., Chow C.R. (2020). HLA associations with infliximab-induced liver injury. Pharmacogenet. J..

[32] Fatima R., Bittar K., Aziz M. (2025). Infliximab.

[33] Brun M.K., Bjorlykke K.H., Viken M.K., Stenvik G.E., Klaasen R.A., Gehin J.E., Warren D.J., Sexton J., Sandanger O., Kvien T.K. (2023). HLA-DQ2 is associated with anti-drug antibody formation to infliximab in patients with immune-mediated inflammatory diseases. J. Intern. Med..

[34] Liu M., Degner J., Davis J.W., Idler K.B., Nader A., Mostafa N.M., Waring J.F. (2018). Identification of HLA-DRB1 association to adalimumab immunogenicity. PLoS ONE.

[35] De Simone C., Farina M., Maiorino A., Fanali C., Perino F., Flamini A., Caldarola G., Sgambato A. (2015). TNF-alpha gene polymorphisms can help to predict response to etanercept in psoriatic patients. J. Eur. Acad. Dermatol. Venereol..

[36] Pan A., Gerriets V. (2025). Etanercept.

[37] Viatte S., Plant D., Han B., Fu B., Yarwood A., Thomson W., Symmons D.P., Worthington J., Young A., Hyrich K.L. (2015). Association of HLA-DRB1 haplotypes with rheumatoid arthritis severity, mortality, and treatment response. JAMA.

[38] Guis S., Balandraud N., Bouvenot J., Auger I., Toussirot E., Wendling D., Mattei J.P., Nogueira L., Mugnier B., Legeron P. (2007). Influence of -308 A/G polymorphism in the tumor necrosis factor alpha gene on etanercept treatment in rheumatoid arthritis. Arthritis Rheumatol..

[39] Gonzalez R.D., Small G.W., Green A.J., Akhtari F.S., Havener T.M., Quintanilha J.C.F., Cipriani A.B., Reif D.M., McLeod H.L., Motsinger-Reif A.A. (2023). RYK Gene Expression Associated with Drug Response Variation of Temozolomide and Clinical Outcomes in Glioma Patients. Pharmaceuticals.

[40] Zhang Z., Wang L., Wei S., Liu Z., Wang L.E., Sturgis E.M., Wei Q. (2010). Polymorphisms of the DNA repair gene MGMT and risk and progression of head and neck cancer. DNA Repair.

[41] Guerra G., Wendt G., McCoy L., Hansen H.M., Kachuri L., Molinaro A.M., Rice T., Guan V., Capistrano L., Hsieh A. (2025). Functional germline variants in DNA damage repair pathways are associated with altered survival in adults with glioma treated with temozolomide. Neuro Oncol..

[42] Carter T., Valenzuela R.K., Yerukala Sathipati S., Medina-Flores R. (2023). Gene signatures associated with prognosis and chemotherapy resistance in glioblastoma treated with temozolomide. Front. Genet..

[43] Colwell M.L., Townsel C., Petroff R.L., Goodrich J.M., Dolinoy D.C. (2023). Epigenetics and the Exposome: DNA Methylation as a Proxy for Health Impacts of Prenatal Environmental Exposures. Exposome.

[44] Huang Y., Wang J., Jiang K., Chung E.J. (2021). Improving kidney targeting: The influence of nanoparticle physicochemical properties on kidney interactions. J. Control. Release.

[45] Chen W., Wang B., Liang S., Wang M., Zheng L., Xu S., Wang J., Fang H., Yang P., Feng W. (2023). Renal clearance of graphene oxide: Glomerular filtration or tubular secretion and selective kidney injury association with its lateral dimension. J. Nanobiotechnol..

[46] Soltysova A., Begerova P., Jakic K., Kozics K., Sramkova M., Meese E., Smolkova B., Gabelova A. (2023). Genome-wide DNA methylome and transcriptome changes induced by inorganic nanoparticles in human kidney cells after chronic exposure. Cell Biol. Toxicol..

[47] Qu L., Yin T., Zhao Y., Lv W., Liu Z., Chen C., Liu K., Shan S., Zhou R., Li X. (2023). Histone demethylases in the regulation of immunity and inflammation. Cell Death Discov..

[48] Abdelhalim M.A. (2011). Exposure to gold nanoparticles produces cardiac tissue damage that depends on the size and duration of exposure. Lipids Health Dis..

[49] Liu G.W., Pippin J.W., Eng D.G., Lv S., Shankland S.J., Pun S.H. (2020). Nanoparticles exhibit greater accumulation in kidney glomeruli during experimental glomerular kidney disease. Physiol. Rep..

[50] Tabish A.M., Poels K., Byun H.M., Luyts K., Baccarelli A.A., Martens J., Kerkhofs S., Seys S., Hoet P., Godderis L. (2017). Changes in DNA Methylation in Mouse Lungs after a Single Intra-Tracheal Administration of Nanomaterials. PLoS ONE.

[51] Yuan Y.G., Zhang Y.X., Liu S.Z., Reza A., Wang J.L., Li L., Cai H.Q., Zhong P., Kong I.K. (2023). Multiple RNA Profiling Reveal Epigenetic Toxicity Effects of Oxidative Stress by Graphene Oxide Silver Nanoparticles in-vitro. Int. J. Nanomed..

[52] Kunovac A., Hathaway Q.A., Pinti M.V., Goldsmith W.T., Durr A.J., Fink G.K., Nurkiewicz T.R., Hollander J.M. (2019). ROS promote epigenetic remodeling and cardiac dysfunction in offspring following maternal engineered nanomaterial (ENM) exposure. Part. Fibre Toxicol..

[53] Gamberoni F., Borgese M., Pagiatakis C., Armenia I., Grazu V., Gornati R., Serio S., Papait R., Bernardini G. (2023). Iron Oxide Nanoparticles with and without Cobalt Functionalization Provoke Changes in the Transcription Profile via Epigenetic Modulation of Enhancer Activity. Nano Lett..

[54] Patil N.A., Gade W.N., Deobagkar D.D. (2016). Epigenetic modulation upon exposure of lung fibroblasts to TiO(2) and ZnO nanoparticles: Alterations in DNA methylation. Int. J. Nanomed..

[55] Pogribna M., Word B., Lyn-Cook B., Hammons G. (2022). Effect of titanium dioxide nanoparticles on histone modifications and histone modifying enzymes expression in human cell lines. Nanotoxicology.

[56] Sánchez-Jaramillo E.A., Gasca-Lozano L.E., Vera-Cruz J.M., Hernández-Ortega L.D., Gurrola-Díaz C.M., Bastidas-Ramírez B.E., Vargas-Guerrero B., Mena-Enríquez M., Martínez-Limón F.J., Salazar-Montes A.M. (2022). Nanoparticles Formulation Improves the Antifibrogenic Effect of Quercetin on an Adenine-Induced Model of Chronic Kidney Disease. Int. J. Mol. Sci..

[57] Paul P., Chacko L., Dua T.K., Chakraborty P., Paul U., Phulchand V.V., Jha N.K., Jha S.K., Kandimalla R., Dewanjee S. (2023). Nanomedicines for the management of diabetic nephropathy: Present progress and prospects. Front. Endocrinol..

[58] Sooklert K., Nilyai S., Rojanathanes R., Jindatip D., Sae-Liang N., Kitkumthorn N., Mutirangura A., Sereemaspun A. (2019). N-acetylcysteine reverses the decrease of DNA methylation status caused by engineered gold, silicon, and chitosan nanoparticles. Int. J. Nanomed..

[59] Wright M.L., Starkweather A.R., York T.P. (2016). Mechanisms of the Maternal Exposome and Implications for Health Outcomes. Adv. Nurs. Sci..

[60] Kimble J., Nusslein-Volhard C. (2022). The great small organisms of developmental genetics: Caenorhabditis elegans and Drosophila melanogaster. Dev. Biol..

[61] Sparrow D.B., Chapman G., Smith A.J., Mattar M.Z., Major J.A., O’Reilly V.C., Saga Y., Zackai E.H., Dormans J.P., Alman B.A. (2012). A mechanism for gene-environment interaction in the etiology of congenital scoliosis. Cell.

[62] Fonseca M.I., Lorigo M., Cairrao E. (2023). Evaluation of the bisphenol A-induced vascular toxicity on human umbilical artery. Environ. Res..

[63] Ye Y., Tang Y., Xiong Y., Feng L., Li X. (2019). Bisphenol A exposure alters placentation and causes preeclampsia-like features in pregnant mice involved in reprogramming of DNA methylation of WNT2. FASEB J..

[64] Muller J.E., Meyer N., Santamaria C.G., Schumacher A., Luque E.H., Zenclussen M.L., Rodriguez H.A., Zenclussen A.C. (2018). Bisphenol A exposure during early pregnancy impairs uterine spiral artery remodeling and provokes intrauterine growth restriction in mice. Sci. Rep..

[65] Cao Y., Chen S., Lu J., Zhang M., Shi L., Qin J., Lv J., Li D., Ma L., Zhang Y. (2023). BPA induces placental trophoblast proliferation inhibition and fetal growth restriction by inhibiting the expression of SRB1. Environ. Sci. Pollut. Res. Int..

[66] Wang Z., Alderman M.H., Asgari C., Taylor H.S. (2020). Fetal Bisphenol-A Induced Changes in Murine Behavior and Brain Gene Expression Persisted in Adult-aged Offspring. Endocrinology.

[67] Sugiyama K.I., Kinoshita M., Gruz P., Kasamatsu T., Honma M. (2022). Bisphenol-A reduces DNA methylation after metabolic activation. Genes Environ..

[68] Fujioka K., Nishida K., Ashina M., Abe S., Fukushima S., Ikuta T., Ohyama S., Morioka I., Iijima K. (2019). DNA methylation of the Rtl1 promoter in the placentas with fetal growth restriction. Pediatr. Neonatol..

[69] Kadan G., Aral N. (2021). Effects of Mycotoxins on Child Development. Curr. Mol. Pharmacol..

[70] Mesfin A., Lachat C., Gebreyesus S.H., Roro M., Tesfamariam K., Belachew T., De Boevre M., De Saeger S. (2023). Mycotoxins Exposure of Lactating Women and Its Relationship with Dietary and Pre/Post-Harvest Practices in Rural Ethiopia. Toxins.

[71] Das Trisha A., Hafsa J.M., Hasan A., Habib A., Tuba H.R., Degen G.H., Ali N. (2024). Occurrence of ochratoxin A in breast milk and urine samples of nursing mothers in Bangladesh. Mycotoxin Res..

[72] Wang J.S., Groopman J.D. (1999). DNA damage by mycotoxins. Mutat. Res..

[73] Feng W.H., Xue K.S., Tang L.L., Williams P.L., Wang J.S. (2017). Aflatoxin B -Induced Developmental and DNA Damage in. Toxins.

[74] Martin J.L., Lin M.Z., McGowan E.M., Baxter R.C. (2009). Potentiation of Growth Factor Signaling by Insulin-like Growth Factor-binding Protein-3 in Breast Epithelial Cells Requires Sphingosine Kinase Activity. J. Biol. Chem..

[75] Erceg S., Mateo E.M., Zipancic I., Rodriguez Jimenez F.J., Perez Arago M.A., Jimenez M., Soria J.M., Garcia-Esparza M.A. (2019). Assessment of Toxic Effects of Ochratoxin A in Human Embryonic Stem Cells. Toxins.

[76] Gromadzka K., Pankiewicz J., Beszterda M., Paczkowska M., Nowakowska B., Kocylowski R. (2021). The Presence of Mycotoxins in Human Amniotic Fluid. Toxins.

[77] Lauer J.M., Duggan C.P., Ausman L.M., Griffiths J.K., Webb P., Wang J.S., Xue K.S., Agaba E., Nshakira N., Ghosh S. (2019). Maternal aflatoxin exposure during pregnancy and adverse birth outcomes in Uganda. Matern. Child Nutr..

[78] Hernandez-Vargas H., Castelino J., Silver M.J., Dominguez-Salas P., Cros M.P., Durand G., Le Calvez-Kelm F., Prentice A.M., Wild C.P., Moore S.E. (2015). Exposure to aflatoxin B1 in utero is associated with DNA methylation in white blood cells of infants in The Gambia. Int. J. Epidemiol..

[79] Owolabi I.O., Siwarak K., Greer B., Rajkovic A., Dall’asta C., Karoonuthaisiri N., Uawisetwathana U., Elliott C.T., Petchkongkaew A. (2023). Applications of Mycotoxin Biomarkers in Human Biomonitoring for Exposome-Health Studies: Past, Present, and Future. Expo. Health.

[80] Eeza M.N.H., Bashirova N., Zuberi Z., Matysik J., Berry J.P., Alia A. (2022). An integrated systems-level model of ochratoxin A toxicity in the zebrafish (*Danio rerio*) embryo based on NMR metabolic profiling. Sci. Rep..

[81] Marin-Kuan M., Ehrlich V., Delatour T., Cavin C., Schilter B. (2011). Evidence for a role of oxidative stress in the carcinogenicity of ochratoxin a. J. Toxicol..

[82] Loboda A., Stachurska A., Podkalicka P., Sobczak M., Mucha O., Witalisz-Siepracka A., Jozkowicz A., Dulak J. (2017). Effect of heme oxygenase-1 on ochratoxin A-induced nephrotoxicity in mice. Int. J. Biochem. Cell Biol..

[83] Klibaner-Schiff E., Simonin E.M., Akdis C.A., Cheong A., Johnson M.M., Karagas M.R., Kirsh S., Kline O., Mazumdar M., Oken E. (2024). Environmental exposures influence multigenerational epigenetic transmission. Clin. Epigenetics.

[84] Babayan N., Tadevosyan G., Khondkaryan L., Grigoryan R., Sarkisyan N., Haroutiounian R., Stopper H. (2020). Ochratoxin A induces global DNA hypomethylation and oxidative stress in neuronal cells in vitro. Mycotoxin Res..

[85] Cui F., Tang L., Li D., Ma Y., Wang J., Xie J., Su B., Tian Y., Zheng X. (2024). Early-life exposure to tobacco, genetic susceptibility, and accelerated biological aging in adulthood. Sci. Adv..

[86] López-Otín C., Blasco M.A., Partridge L., Serrano M., Kroemer G. (2013). The Hallmarks of Aging. Cell.

[87] Pal S., Tyler J.K. (2016). Epigenetics and aging. Sci. Adv..

[88] Zhuang X., Wang Q., Joost S., Ferrena A., Humphreys D.T., Li Z., Blum M., Krause K., Ding S., Landais Y. (2025). Ageing limits stemness and tumorigenesis by reprogramming iron homeostasis. Nature.

[89] Gendron C.M., Chakraborty T.S., Duran C., Dono T., Pletcher S.D. (2023). Ring neurons in the Drosophila central complex act as a rheostat for sensory modulation of aging. PLoS Biol..

[90] Hara T., Asatsu M., Yamagishi T., Ohata C., Funatsu H., Takahashi Y., Shirai M., Nakata C., Katayama H., Kaji T. (2024). Cadmium Induces Vascular Endothelial Cell Detachment by Downregulating Claudin-5 and ZO-1 Levels. Int. J. Mol. Sci..

[91] Almeida-Silva M., Cardoso J., Alemao C., Santos S., Monteiro A., Manteigas V., Marques-Ramos A. (2022). Impact of Particles on Pulmonary Endothelial Cells. Toxics.

[92] Pandics T., Major D., Fazekas-Pongor V., Szarvas Z., Peterfi A., Mukli P., Gulej R., Ungvari A., Fekete M., Tompa A. (2023). Exposome and unhealthy aging: Environmental drivers from air pollution to occupational exposures. Geroscience.

[93] Pan Z., Gong T., Liang P. (2024). Heavy Metal Exposure and Cardiovascular Disease. Circ. Res..

[94] Moutsianas L., Jostins L., Beecham A.H., Dilthey A.T., Xifara D.K., Ban M., Shah T.S., Patsopoulos N.A., Alfredsson L., Anderson C.A. (2015). Class II HLA interactions modulate genetic risk for multiple sclerosis. Nat. Genet..

[95] Matsuzaki R., Gunnigle E., Geissen V., Clarke G., Nagpal J., Cryan J.F. (2023). Pesticide exposure and the microbiota-gut-brain axis. ISME J..

[96] Elsayed N.S., Valenzuela R.K., Kitchner T., Le T., Mayer J., Tang Z.Z., Bayanagari V.R., Lu Q., Aston P., Anantharaman K. (2023). Genetic risk score in multiple sclerosis is associated with unique gut microbiome. Sci. Rep..

[97] Nasr Z., Schoeps V.A., Ziaei A., Virupakshaiah A., Adams C., Casper T.C., Waltz M., Rose J., Rodriguez M., Tillema J.M. (2023). Gene-environment interactions increase the risk of paediatric-onset multiple sclerosis associated with household chemical exposures. J. Neurol. Neurosurg. Psychiatry.

[98] Zhou Y., Cui C., Ma X., Luo W., Zheng S.G., Qiu W. (2020). Nuclear Factor kappaB (NF-kappaB)-Mediated Inflammation in Multiple Sclerosis. Front. Immunol..

[99] Pamphlett R., Buckland M.E., Bishop D.P. (2023). Potentially toxic elements in the brains of people with multiple sclerosis. Sci. Rep..

[100] Goutman S.A., Savelieff M.G., Jang D.G., Hur J., Feldman E.L. (2023). The amyotrophic lateral sclerosis exposome: Recent advances and future directions. Nat. Rev. Neurol..

[101] Garretti F., Monahan C., Sloan N., Bergen J., Shahriar S., Kim S.W., Sette A., Cutforth T., Kanter E., Agalliu D. (2023). Interaction of an α-synuclein epitope with HLA-DRB1(∗)15:01 triggers enteric features in mice reminiscent of prodromal Parkinson’s disease. Neuron.

[102] Lopez-Pingarron L., Almeida H., Soria-Aznar M., Reyes-Gonzales M.C., Terron M.P., Garcia J.J. (2023). Role of Oxidative Stress on the Etiology and Pathophysiology of Amyotrophic Lateral Sclerosis (ALS) and Its Relation with the Enteric Nervous System. Curr. Issues Mol. Biol..

[103] Paul K.C., Sinsheimer J.S., Rhodes S.L., Cockburn M., Bronstein J., Ritz B. (2016). Organophosphate Pesticide Exposures, Nitric Oxide Synthase Gene Variants, and Gene-Pesticide Interactions in a Case-Control Study of Parkinson’s Disease, California (USA). Environ. Health Perspect..

[104] da Silva D.R.F., Bittencourt L.O., Aragao W.A.B., Nascimento P.C., Leao L.K.R., Oliveira A.C.A., Crespo-Lopez M.E., Lima R.R. (2020). Long-term exposure to lead reduces antioxidant capacity and triggers motor neurons degeneration and demyelination in spinal cord of adult rats. Ecotoxicol. Environ. Saf..

[105] Xu Z., Hu T., Xu C., Liang X., Li S., Sun Y., Liu F., Wang J., Tang Y. (2024). Disease progression in proposed brain-first and body-first Parkinson’s disease subtypes. npj Park. Dis..

[106] Costa L.G., Cole T.B., Furlong C.E. (2006). Gene-environment interactions: Paraoxonase (PON1) and sensitivity to organophosphate toxicity. Labmedicine.

[107] Li W.F., Costa L.G., Richter R.J., Hagen T., Shih D.M., Tward A., Lusis A.J., Furlong C.E. (2000). Catalytic efficiency determines the efficacy of PON1 for detoxifying organophosphorus compounds. Pharmacogenetics.

[108] Medina-Diaz I.M., Ponce-Ruiz N., Ramirez-Chavez B., Rojas-Garcia A.E., Barron-Vivanco B.S., Elizondo G., Bernal-Hernandez Y.Y. (2017). Downregulation of human paraoxonase 1 (PON1) by organophosphate pesticides in HepG2 cells. Environ. Toxicol..

[109] Feng J., Zheng Y., Ma W., Weng D., Peng D., Xu Y., Wang Z., Wang X. (2024). A synthetic antibiotic class with a deeply-optimized design for overcoming bacterial resistance. Nat. Commun..

[110] Schambach A., Buchholz C.J., Torres-Ruiz R., Cichutek K., Morgan M., Trapani I., Buning H. (2024). A new age of precision gene therapy. Lancet.

[111] Laurent M., Geoffroy M., Pavani G., Guiraud S. (2024). CRISPR-Based Gene Therapies: From Preclinical to Clinical Treatments. Cells.

[112] Zhang R., Yan X., Gao L., Fan K. (2025). Nanozymes expanding the boundaries of biocatalysis. Nat. Commun..

[113] Hynek N. (2025). Synthetic biology/AI convergence (SynBioAI): Security threats in frontier science and regulatory challenges. Ai Soc..

[114] Wittmann B.J., Alexanian T., Bartling C., Beal J., Clore A., Diggans J., Flyangolts K., Gemler B.T., Mitchell T., Murphy S.T. (2025). Strengthening nucleic acid biosecurity screening against generative protein design tools. Science.

[115] Hoffmann S.A., Diggans J., Densmore D., Dai J., Knight T., Leproust E., Boeke J.D., Wheeler N., Cai Y. (2023). Safety by design: Biosafety and biosecurity in the age of synthetic genomics. iScience.

[116] Lovato T.L., Blotz B., Bileckyj C., Johnston C.A., Cripps R.M. (2023). Modeling a variant of unknown significance in the Drosophila ortholog of the human cardiogenic gene NKX2.5. Dis. Model. Mech..

[117] Platt R.J., Chen S., Zhou Y., Yim M.J., Swiech L., Kempton H.R., Dahlman J.E., Parnas O., Eisenhaure T.M., Jovanovic M. (2014). CRISPR-Cas9 knockin mice for genome editing and cancer modeling. Cell.

[118] Musunuru K., Grandinette S.A., Wang X., Hudson T.R., Briseno K., Berry A.M., Hacker J.L., Hsu A., Silverstein R.A., Hille L.T. (2025). Patient-Specific In Vivo Gene Editing to Treat a Rare Genetic Disease. N. Engl. J. Med..

[119] Siegert M.J., Knittel C.H., Sussmuth R.D. (2020). A Convergent Total Synthesis of the Death Cap Toxin alpha-Amanitin. Angew. Chem. Int. Ed. Engl..

[120] Le Dare B., Ferron P.J., Gicquel T. (2021). Toxic Effects of Amanitins: Repurposing Toxicities toward New Therapeutics. Toxins.

[121] Mathur M., Beniwal P., Prasad D., Malhotra V. (2014). Occupational kidney disease: Is exposure to ammonium nitrate a risk factor?. Indian J. Occup. Environ. Med..

[122] DeTata D., D’Uva J.A., Lewis S.W. (2022). Source determination of homemade ammonium nitrate using ATR-FTIR spectroscopy, trace elemental analysis and chemometrics. Forensic Chem..

[123] U.S. Department of Health and Human Services (2007). Toxicological Profile for Benzene. Agency for Toxic Substances and Disease Registry (ATSDR) Toxicological Profiles.

[124] Angelini M., Seyyedsalehi M.S., Boffetta P. (2025). Occupational benzene exposure and skin cancers: A systematic review and meta-analysis. Occup. Med..

[125] Hahladakis J.N., Iacovidou E., Gerassimidou S. (2023). An overview of the occurrence, fate, and human risks of the bisphenol-A present in plastic materials, components, and products. Integr. Environ. Assess. Manag..

[126] Vinod V., Amritha P.S., Harathi P.B. (2023). A systematic review on bisphenols-Sources, health impacts, and analytical determination of bisphenol A in aqueous samples. Sep. Sci. Plus.

[127] Murjani B.O., Kadu P.S., Bansod M., Vaidya S.S., Yadav M.D. (2022). Carbon nanotubes in biomedical applications: Current status, promises, and challenges. Carbon Lett..

[128] Brito C.L., Silva J.V., Gonzaga R.V., La-Scalea M.A., Giarolla J., Ferreira E.I. (2024). A Review on Carbon Nanotubes Family of Nanomaterials and Their Health Field. ACS Omega.

[129] U.S. Department of Health and Human Services (2005). Toxicological Profile for Carbon Tetrachloride. Agency for Toxic Substances and Disease Registry (ATSDR) Toxicological Profiles.

[130] Cohen S.M., Bevan C., Gollapudi B., Klaunig J.E. (2023). Evaluation of the carcinogenicity of carbon tetrachloride. J. Toxicol. Environ. Health B Crit. Rev..

[131] Garrison A.R., Moresco V., Zeng X., Cline C.R., Ward M.D., Ricks K.M., Olschner S.P., Cazares L.H., Karaaslan E., Fitzpatrick C.J. (2024). Nucleocapsid protein-specific monoclonal antibodies protect mice against Crimean-Congo hemorrhagic fever virus. Nat. Commun..

[132] Swanepoel R., Gill D.E., Shepherd A.J., Leman P.A., Mynhardt J.H., Harvey S. (1989). The clinical pathology of Crimean-Congo hemorrhagic fever. Rev. Infect. Dis..

[133] Hawman D.W., Feldmann H. (2023). Crimean-Congo haemorrhagic fever virus. Nat. Rev. Microbiol..

[134] Marques M.L., Cairrao E. (2023). Occurrence and Health Effects of Hexabromocyclododecane: An Updated Review. Toxics.

[135] Marvin C.H., Tomy G.T., Armitage J.M., Arnot J.A., McCarty L., Covaci A., Palace V. (2011). Hexabromocyclododecane: Current understanding of chemistry, environmental fate and toxicology and implications for global management. Environ. Sci. Technol..

[136] Li Y., Wang L., Shi L., Lou Y., Liu A., Lin Y., Yang R., Gao W., Qu G. (2025). Bioaccumulation and Maternal Transfer of Brominated Flame Retardants in Poultry and the Health Risks from Dietary Exposure. Environ. Health.

[137] Jasani S. (2015). Smoke inhalation. Small Animal Critical Care Medicine.

[138] Das S. (2020). Toxic gases. Toxicology Cases for the Clinical and Forensic Laboratory.

[139] Li C., Srivastava R.K., Athar M. (2016). Biological and environmental hazards associated with exposure to chemical warfare agents: Arsenicals. Ann. N. Y. Acad. Sci..

[140] Watson A.P., Griffin G.D. (1992). Toxicity of vesicant agents scheduled for destruction by the Chemical Stockpile Disposal Program. Environ. Health Perspect..

[141] Srivastava R.K., Muzaffar S., Khan J., Traylor A.M., Zmijewski J.W., Curtis L.M., George J.F., Ahmad A., Antony V.B., Agarwal A. (2020). Protective role of HO-1 against acute kidney injury caused by cutaneous exposure to arsenicals. Ann. N. Y. Acad. Sci..

[142] IARC (2012). Chemical Agents and Related Occupations.

[143] Armoo A., Diemer T., Donkor A., Fedorchik J., Van Slambrouck S., Willand-Charnley R., Logue B.A. (2023). Methimazole, an Effective Neutralizing Agent of the Sulfur Mustard Derivative 2-Chloroethyl Ethyl Sulfide. ACS Bio Med Chem Au.

[144] Jowsey P.A., Williams F.M., Blain P.G. (2009). DNA damage, signalling and repair after exposure of cells to the sulphur mustard analogue 2-chloroethyl ethyl sulphide. Toxicology.

[145] Malaviya R., Laskin J.D., Laskin D.L. (2020). Long-term Respiratory Effects of Mustard Vesicants. Toxicol. Lett..

[146] Szoke Z., Babarczi B., Mezes M., Lakatos I., Poor M., Fliszar-Nyul E., Oldal M., Czeh A., Bodo K., Nagyeri G. (2022). Analysis and Comparison of Rapid Methods for the Determination of Ochratoxin a Levels in Organs and Body Fluids Obtained from Exposed Mice. Toxins.

[147] Bui-Klimke T.R., Wu F. (2015). Ochratoxin A and human health risk: A review of the evidence. Crit. Rev. Food Sci. Nutr..

[148] el Khoury A., Atoui A. (2010). Ochratoxin a: General overview and actual molecular status. Toxins.

[149] Wieckowska M., Szelenberger R., Niemcewicz M., Harmata P., Poplawski T., Bijak M. (2023). Ochratoxin A-The Current Knowledge Concerning Hepatotoxicity, Mode of Action and Possible Prevention. Molecules.

[150] Robb E.L., Regina A.C., Baker M.B. (2025). Organophosphate Toxicity.

[151] Boczkowski M., Popiel S., Nawala J., Suska H. (2025). History of Organophosphorus Compounds in the Context of Their Use as Chemical Warfare Agents. Molecules.

[152] Bast C.B., Glass-Mattie D.F. (2020). Phosgene. Handbook of Toxicology of Chemical Warfare Agents.

[153] Renzelli V., Gallo M., Morviducci L., Marino G., Ragni A., Tuveri E., Faggiano A., Mazzilli R., Natalicchio A., Zatelli M.C. (2023). Polybrominated Diphenyl Ethers (PBDEs) and Human Health: Effects on Metabolism, Diabetes and Cancer. Cancers.

[154] Hayoun M.A., Kong E.L., Smith M.E., King K.C. (2025). Ricin Toxicity.

[155] Bolt H.M., Hengstler J.G. (2023). Ricin: An ancient toxicant, but still an evergreen. Arch. Toxicol..

[156] Reisler R.B., Smith L.A. (2012). The need for continued development of ricin countermeasures. Adv. Prev. Med..

[157] Chen Z., Zakrzewska S., Hajare H.S., Alvarez-Buylla A., Abderemane-Ali F., Bogan M., Ramirez D., O’Connell L.A., Du Bois J., Minor D.L. (2022). Definition of a saxitoxin (STX) binding code enables discovery and characterization of the anuran saxiphilin family. Proc. Natl. Acad. Sci. USA.

[158] Cusick K.D., Sayler G.S. (2013). An overview on the marine neurotoxin, saxitoxin: Genetics, molecular targets, methods of detection and ecological functions. Mar. Drugs.

[159] Bick A.G., Metcall G.A., Mayo K.R., Lichtenstein L., Rura S., Carroll R.J., Musick A., Linder J.E., Jordan I.K., Nagar S.D. (2024). Genomic data in the All of Us Research Program. Nature.

[160] Safarlou C.W., Jongsma K.R., Vermeulen R., Bredenoord A.L. (2023). The ethical aspects of exposome research: A systematic review. Exposome.

[161] Coughlin S.S., Dawson A. (2014). Ethical, Legal and Social Issues in Exposomics: A Call for Research Investment. Public Health Ethics.

[162] Martin-Sanchez F.J., Lopez-Campos G.H. (2016). The New Role of Biomedical Informatics in the Age of Digital Medicine. Method. Inform. Med..

